# Sedation for Patients with Sepsis: Towards a Personalised Approach

**DOI:** 10.3390/jpm13121641

**Published:** 2023-11-24

**Authors:** José Miguel Marcos-Vidal, Rafael González, María Merino, Eva Higuera, Cristina García

**Affiliations:** Department of Anesthesiology and Critical Care, Universitary Hospital of Leon, 24071 Leon, Spain; rgonzalezdc@saludcastillayleon.es (R.G.); mmerinoga@saludcastillayleon.es (M.M.); emhiguera@saludcastillayleon.es (E.H.); cgarciaper@saludcastillayleon.es (C.G.)

**Keywords:** sedation, intensive care unit, sepsis

## Abstract

This article looks at the challenges of sedoanalgesia for sepsis patients, and argues for a personalised approach. Sedation is a necessary part of treatment for patients in intensive care to reduce stress and anxiety and improve long-term prognoses. Sepsis patients present particular difficulties as they are at increased risk of a wide range of complications, such as multiple organ failure, neurological dysfunction, septic shock, ARDS, abdominal compartment syndrome, vasoplegic syndrome, and myocardial dysfunction. The development of any one of these complications can cause the patient’s rapid deterioration, and each has distinct implications in terms of appropriate and safe forms of sedation. In this way, the present article reviews the sedative and analgesic drugs commonly used in the ICU and, placing special emphasis on their strategic administration in sepsis patients, develops a set of proposals for sedoanalgesia aimed at improving outcomes for this group of patients. These proposals represent a move away from simplistic approaches like avoiding benzodiazepines to more “objective-guided sedation” that accounts for a patient’s principal pathology, as well as any comorbidities, and takes full advantage of the therapeutic arsenal currently available to achieve personalised, patient-centred treatment goals.

## 1. Introduction

Sedation for critically ill patients for is a complex and fast-changing field of medicine. Several recent publications have raised questions about some of the most widely used sedatives, for example, propofol [1] and single doses of etomidate [2]. In the case of the latter, concerns about adverse effects were such that it has been removed from the arsenal of drugs used for prolonged sedation. At the same time, there is an ongoing trend to limit the use of opioids due to their harmful side effects [3,4], while environmental concerns are increasingly sidelining once promising research into fluorinated inhalation sedatives [5].

Of course, the necessity of sedation is indisputable, particularly for patients in intensive care units (ICUs). These patients are likely to be experiencing pain, agitation, and anxiety due to their being subject to variously invasive monitoring and other procedures, most notably mechanical ventilation. The sedation of ICU patients should have several simultaneous objectives, including minimizing their oxygen consumption, maintaining them comfortably connected to the ventilator, and generally reducing stress and anxiety to facilitate any necessary procedures while, at the same time, avoiding any clinical deterioration in the patient’s condition and preventing either physiological or psychological harm. Achieving all these goals is far from trivial in everyday practice, and especially during the initial phases of recovery for septic surgical or neurocritical patients, or in those with associated intra-abdominal hypertension or acute respiratory distress syndrome (ARDS).

Among critically ill patients, those with sepsis present with a very particular set of issues [6]. Dysregulated systemic inflammation due to an underlying infection may cause the clinical syndrome of sepsis or septic shock, defined as a “life-threatening organ dysfunction due to a dysregulated host response to infection” in the Sepsis-3 definition [7]. Sepsis is characterised by the widespread release of cytokines, proteases, and reactive oxygen species, which lead to both direct and indirect cellular damage. Vasodilatation and capillary leak ensue, resulting in relative and absolute intravascular hypovolaemia in the first instance, followed by the potential for considerable increase in total body fluid after resuscitation. Microcirculatory blood flow is impaired, leading to heterogeneous organ perfusion, mitochondrial dysfunction, cellular hypoxia, and subsequently organ dysfunction and failure. The extent to which these changes occur depends on the complex interplay between infectious factors, patient factors, and treatment factors [8].

The basic approach towards sepsis can be summarised as timely recognition, the annihilation of infection including source control and early initiation of adequate antimicrobial therapy, volume resuscitation, and vasopressor and treatment in case of shock. Beyond this, clinicians face the spectre of multi-organ dysfunction syndrome (MODS). MODS is not a single event but a continuum of processes characterised by serial and incremental physiologic assaults on individual organs. Virtually all organs are involved, but damage may vary from hardly detectable or mild to completely irreversible [9]. One of the many fascinating paradoxes of sepsis is its variable effect on the body’s organs. The most frequently affected organs are the lungs (with ARDS as the most severe form of affectation), brain (with clinical features of encephalopathy, including agitation, confusion, and coma), hepatosplanchnic system (clinical features of liver dysfunction generally occur later in the septic process and, if present, portend a worse outcome), kidney (acute kidney injury) and heart (cardiomyopathy) [10].

Sepsis causes several pathophysiological changes that alter drug disposition, the effects of which can be significant. The redistribution of blood away from peripheral tissues with or without a reduction in cardiac output can decrease the volume of distribution of some fat-soluble medications, leading to higher plasma concentrations. This is particularly important in critical care for rapidly acting medications with concentration-dependent adverse effects, such as intravenous anaesthetic, analgesic, or sedative agents [11].

This means that there are numerous factors that need to be considered when selecting the best sedoanalgesic approach for each patient, for instance, the cardiovascular effects of a given drug, potentially variable pharmacokinetics or pharmacodynamics, or the possibility of drug interactions in polymedicated patients. Thus, all possible combinations of drug alternatives must be assessed with the objective of preventing both the physiological and psychological harms associated with critical care. Furthermore, considering sepsis patients in particular, it must be stressed that each individual will have a unique clinical context, thus, a personalised therapeutic approach is essential.

In the present article, we review the principal characteristics of the sedative and analgesic drugs most widely used in ICUs, placing special emphasis on their strategic administration and monitoring in sepsis patients. In this way, we develop a set of intervention proposals addressing the objectives of sedation mentioned above, and the particular needs of this group of patients.

## 2. Monitoring Sedoanalgesia

The effective administration and management of sepsis patients requires the subjective and/or objective monitoring of three key variables: pain, agitation, and the level of consciousness (LOC). In assessing these three variables, it is essential (1) to have a set of predetermined goals [12]; (2) to employ tools that are not only valid and precise but also easy to implement, such that they can be deployed flexibly (including at least one assessment per shift); (3) and to provide defined protocols for the evaluation and management of analgesia, sedation, and delirium [13,14]. To this end, and to facilitate the multimodal approach necessary for critically ill patients, certain initiatives have been proposed, for instance the ABCDEF (A2F) bundle (Assess, prevent, and manage pain; Both spontaneous awakening and breathing trials; Choice of analgesia and sedation; Delirium assess, prevent, and manage; Early mobility and exercise; Family engagement/empowerment) [15]. This bundle is designed to ensure ICU patients receive wholistic care, better controlled pain and, importantly, regain higher-order physical and mental capacities early on in their recovery from critical illness. Another initiative involves the so-called eCASH approach [16] (early Comfort using Analgesia, minimal Sedatives and maximal Humane care), which proposes the integration of light sedation, among a range of measures primarily aimed at targeting pain and promoting sleep. In both cases, the A2F bundle and the eCASH approach, appropriate tools exist to evaluate each element of the relevant protocol.

### 2.1. Evaluating and Monitoring Pain

To assess levels of pain and thus analgesic requirements, where possible, it is always recommended to use a scale based on self-reported information from the patient. To this end, two such scales exist: the Visual Numeric Scale (VNS) and the Visual Analogue Scale (VAS), where the VNS has a lower incidence of non-response (2%) compared to the VAS (11%) [17]. In cases where the patient is unable to provide the requisite information themselves, it is possible to refer to physiological and behavioural pain indicators using the Behaviour Pain Scale (BPS) and the Critical Care Observation Tool (CPOT) [18]. One limitation of the BPS is the observation that sedation reduces responsivity, and this particularly affects the dimension of compliance with mechanical ventilation leaving only facial expressions and movement in the upper extremities with which to evaluate pain.

Scales have also been developed specifically for pain assessment in paediatric patients (COMFORT, FLACC), and there are several others that are rarely used due to their complexity and the fact that they provide little additional benefit in the evaluation of pain in ICU contexts [19]. Furthermore, while currently not recommended in any existing guidelines for the management of critically ill patients, in recent years, various methods have been developed to monitor nociceptive response during surgical procedures, for example, the Nociception Level Index (NOL), the Med-Storm Innovation AS, pupillometry, the Cardiac Index, and the Analgesia Nociception Index (ANI) [20].

### 2.2. Evaluating and Monitoring Sedation

With respect to sedation monitoring, the two scales that are both most widely used and recommended in available clinical practice guides are the Sedation-Agitation Scale (SAS) and the Richmond Agitation Sedation Scale (RASS) are. This is due to their excellent reliability and interrater concordance.

Where sedation is achieved by inhalation of halogenated agents, the concentration of the inhaled substance in exhaled gases may be monitored using the appropriate device and the value obtained compared to the minimum alveolar concentration (MAC) for the anaesthetic in question. Several MAC values are defined; however, that used to dose sedatives in ICU is MAC-awake, and in the case of sevoflurane this value is between 0.5 and 1%. For a given sedative, MAC values decrease with the age of the patient and where it is used concurrently with additional sedatives or opioids [21].

### 2.3. Diagnosis of Delirium

The clinical diagnosis of delirium is made using one of several validated scales, for instance, the Confusion Assessment Method for the ICU (CAM-ICU) and the Intensive Care Delirium Screening Checklist (ICDSC), both of which can identify patients presenting with forms of hyperactive delirium [22]. An evaluation of delirium should be completed at least once each shift and every time a change in the patient’s LOC is noted (for example, before and after the cessation of sedation). The LOC is an important factor in assessing delirium; thus, optimum levels of sedation must be ensured at all times and when using the CAM-ICU scale for delirium, it can be evaluated with RASS. The ICDSC is considered most appropriate for patients who are unable to communicate as it includes data obtained during routine observations made by the nursing team overseeing patient care.

Three models exist to predict the onset of delirium including the PRE-DELIRIC model; the early prediction model, E-PRE-DELIRIC; and the Lanzhou model. These models are invaluable in enabling medical teams to implement measures, especially non-drug-based interventions [23], to prevent delirium.

The electroencephalogram (EEG) is also a potentially useful tool for the evaluation of delirium. Inflammatory mediators can cross the blood–brain barrier and increase vascular permeability which, in turn, produces changes in brain activity that can be seen on an EEG. However, the difficulties of implementing and interpreting EEG data prevent it from becoming a routine tool in ICU settings. Critical care continuous EEG (CCEEG) refers to the simultaneous recording of EEG and clinical behaviour (video) over extended time periods (hours to weeks) in critically ill patients. CCEEG is usually performed in an ICU setting, but this varies by hospital, and some patients may be in step-down units or general medical or surgical units. The goal of CCEEG is to identify changes in brain function, such as nonconvulsive seizures or ischemia, which may not be evident using neurological examination alone, in order to facilitate the early identification and management of these abnormalities [24]. However, in sepsis patients, due to the frequency of delirium [25], the timely use of EEG may be recommended to identify non-convulsive epileptic seizures, which may be indicative of delirium.

The current literature strongly suggests that there is a moderate correlation between bispectral index (BIS) measurements and two clinical scales for assessing sedation: RASS and SAS [26]; thus, BIS may be used in cases where it is impossible to evaluate a patient using these clinical scales. However, some important questions remain regarding the routine use of BIS with patients who have received neuromuscular blocking agents (NMBAs). Specifically, it is unknown what the optimal range of BIS values is in these cases, since NMBAs themselves reduce electromyogram activity, one of the main metrics used to obtain BIS values [27]; thus, BIS is not a reliable indicator of awareness in these patients. In addition, there is a lack of high-quality studies concerning the impact of BIS monitoring on length of stay in ICU or ventilator-free days; hence, it is currently not recommended as a routine tool [28], and the use of clinical scales should be prioritised. Table 1 presents a summary of the different tools currently used in the assessment of pain, sedation, and delirium.

## 3. Sedation via Intravenous Agents

### 3.1. Propofol

Propofol is highly liposoluble and has a very short elimination half-life, making it ideal for delivery via infusion, as well as providing fast recovery from sedation. In contrast to benzodiazepines, which are excreted by the kidneys, propofol metabolism occurs principally via conjugation in the liver (with some extrahepatic clearance in the kidneys) to form inactive metabolites. This means that no dose adjustment is required for patients experiencing renal or hepatic insufficiency. However, in the case of patients with septic shock, liver function is decreased up to threefold; thus, dose accumulation and over-sedation may occur. Indeed, research shows this might happen in up to 60% of ICU admissions. Propofol consists of highly lipid-soluble molecules with extensive protein binding (98% bound to albumin). In severe sepsis, the volume of distribution is initially decreased as a consequence of the centralisation of blood flow. This, combined with a decreased serum albumin, can lead to significantly higher free plasma concentrations, causing pronounced cardiovascular effects. Decreased cardiac output also prolongs the time to induction of anaesthesia, and doses should be reduced, given slowly, and titrated to effect [11].

In addition to the above considerations, propofol’s principal adverse effects are associated with cases of prolonged sedation. The main issues are related the fact that this drug is formulated as a lipid emulsion, and thus long-term use can lead to hypertriglyceridemia; it also means this drug must be considered as a source of calories as part of the nutritional care of critically ill patients [29]. Another potential complication is propofol-related infusion syndrome (PRIS), which, although rare, is potentially lethal, with a mortality rate of around 20–80%. While the presentation of PRIS does not have a standard definition, it is related to high infusion times (>48 h) and doses (>80 µg/kg/min), or accumulated doses (>300 mg/kg), where this last factor is more important that total infusion time [30]. PRIS is more frequent in younger patients, particularly those on catecholamines or corticosteroids and those with severe illness. Unfortunately, it is also true that severe illness can delay or impede the diagnosis of PRIS, especially in cases of septic shock.

Propofol acts by targeting the GABA-A receptors, and the activation of these receptors is associated with sedation-induced delirium. This mechanism is shared with the benzodiazepines; however, the rapid clearance of propofol from the nervous system [31] means that, compared to benzodiazepines, it may result in shorter episodes of delirium.

Here, it is worth paying special attention to the development of intra-abdominal hypertension syndrome (IAH) and its final stage, abdominal compartment syndrome (ACS). Once IAH is diagnosed, it is immediately necessary to implement non-surgical measures to reduce intra-abdominal pressure (IAP); these may include attempting to produce a negative fluid balance through diaresis or dialysis, the evaluation of intra-abdominal content, and optimizing sedation. Although mechanical ventilation should be avoided as far as possible due to its potential for elevating IAP, when it is necessary, the use of deep sedation and even a neuromuscular blockade can help improve abdominal compliance, and thus reduce IAP [32]. In such cases, propofol is the first choice as an intravenous sedative because it reduces breathing drive that would otherwise increase IAP [33]. The use of neuromuscular relaxants is reserved only for cases that do not respond to this treatment and as the last resort before surgical intervention.

### 3.2. Dexmedetomidine

Dexmedetomidine (DEX) is a highly selective alpha2-andrenergic agonist that strongly inhibits the sympathetic nervous system, decreasing the heart rate and myocardial oxygen consumption. Its use can result in a biphasic arterial blood pressure response, particularly in older patients, involving a reduction in arterial pressure at low doses and hypertension in high doses (up to 0.7 µg/kg/h).

The dose required to achieve optimal sympatholytic effects while protecting organ function is unknown. However, it must be noted that liver dysfunction can inhibit the elimination of dexmedetomidine from the body. In addition, it is not recommended as a sedative for patients with severe neurological disorders, especially when in the acute phase, due to the reduction in blood flow to the brain, or for those with disorders of the autonomic nervous system.

The use of DEX is also contraindicated in cases of persistent bradycardia or with the novel appearance of symptoms of second- or third-degree heart block, and indeed, its use should be suspended should these symptoms appear after administration. Similarly, DEX is inadvisable where hypothermia is suspected or develops due to its use. There are not sufficient data to give a maximum time limit for the use of this drug; however, it is recommended that continuous administration should not extend beyond seven days. After prolonged infusion times, sudden cessation can cause withdrawal symptoms, and thus, it is recommended that doses should be reduced gradually.

It is difficult to obtain deep sedation using DEX, which complicates its management in cases of sepsis, particularly in the first 48 h when rescue treatment with propofol or benzodiazepines may be required. However, it is also true that the use of DEX may help reduce the necessary doses of these other medications. Furthermore, DEX improves the quality and duration of sleep without eliminating non-REM sleep, meaning its effects mimic natural sleep and thus generate a stable sleep–wake circadian rhythm. This is helpful for critically ill patients, who often experience poor quality sleep and non-restorative, disorganised sleep patterns. This factor may also contribute to a lower risk of delirium for DEX compared to other sedatives; however, the available evidence is currently inconclusive [34].

### 3.3. Antipsychotics

Despite contradictory evidence concerning their effectiveness against delirium and the identification of significant adverse side-effects in non-critically ill patients, antipsychotic drugs continue to be widely used to treat this condition in ICU settings [35]. Atypical antipsychotics (quetiapine, aripiprazole, olanzapine, and risperidone) have become increasingly popular due to their lower risk of adverse side-effects compared to typical antipsychotics; specifically, they are associated with a lower incidence of cardiac arrythmia, prolonged QT interval, and neuroleptic malignant syndrome, all of which are especially problematic in older patients.

Currently there is some controversy over the need to treat hypoactive delirium, and since the trend is towards the avoidance of drug treatments, non-drug measures alone are recommended [23]. Equally, at present, there are no firm recommendations regarding any form of drug-based treatment for hyperactive delirium. There is sparse evidence that any drug can be said to reduce the incidence of delirium, or indeed the duration of episodes; however, for intubated patients, the use of DEX is a reasonable measure and, in cases of hyperactive delirium, so is combining this drug with antipsychotics. Furthermore, propofol would be the agent of choice in situations requiring the rapid reduction in delirium-associated agitation. Meanwhile, benzodiazepines (especially midazolam and lorazepam) should always be avoided [36].

### 3.4. Analgesic Agents

Pain management in critically ill patients should always be a priority due to the discomfort of prolonged immobilization and the need for various routine procedures in ICU settings that may cause pain, for instance, the insertion of vascular cannulas, insertion and removal of drainage tubes, changes in position, and the aspiration of endotracheal secretions. Indeed, analgesia should even take precedence over choice of sedative. In this respect, opioids continue to be the main pillar of pain treatment, and opium-based analgesics are recommended for use in ICUs. While there has been an increase in the use of synthetic opioids such as remifentanil, which have very short duration of action times, the most-used opioids among critically ill patients are morphine and fentanyl, each of which has very different pharmacological properties. Of these two, fentanyl has a shorter onset of action time; it is also rapidly transported to fat-rich body tissues and metabolised in the liver. It has a context-sensitive half-life, with its effects being extended when infused for long periods. In contrast, morphine is rapidly broken down into active metabolites that can accumulate, particularly in cases of renal dysfunction, thus prolonging its active effects. There are few studies comparing these two opioids; however, there appears to be little difference in terms of ventilator-free days or the length of stay in ICUs [37]. Nevertheless, there are reasons to favour fentanyl for patients with creatine levels > 1.7 mg/dL. Elevated creatine levels are a frequent finding in sepsis patients with associated kidney damage, and in such cases, intravenous morphine should be used with caution. On the other hand, fentanyl tends to have a reduced elimination rate and longer elimination half-life in older patients, which has repercussions for ventilator-free days.

Remifentanil is the opioid most consistently associated with the development of opioid-induced hyperalgesia (OIH) [38], a condition that must be distinguished from acute opioid withdrawal, and which presents particularly where opioid drugs have been used at high dosages over long time periods with an especially elevated incidence in younger patients. This condition may require the use of additional analgesics, and as a result may prolong the time spent on mechanical ventilation. The onset of OIH tends to occur 45 min after drug withdrawal and, in most cases, is only a significant issue during the first few hours of drug withdrawal. However, cases have been reported where increased pain sensitivity has continued for as long as three months. Remifentanil can be considered as a treatment for patients with renal insufficiency, liver failure, and in neurocritical patients to facilitate neurological examination.

Once again, a multimodal approach helps to diminish the use of opioids. In this way, paracetamol is recommended as an adjuvant to opioids for the treatment of post-operative pain, especially after abdominal surgery where there is risk to the ilium or the presence of nausea and/or vomiting. In sepsis patients, paracetamol’s antipyretic effects can be useful in some cases, but care must be taken as its administration may obscure the presence of febrile peaks. Gabapentin, carbamazepine, and pregabalin are recommended for cases of neuropathic pain; however, due precaution must be taken to avoid elevated doses in cases of renal insufficiency and because of their potential effects on cognition and sedation in some patients. Other adjuvant therapies are not recommended, including, for instance, COX-1 selective NSAIDs (due to enhanced risk of bleeding and kidney injury), ketamine (due to potential neurotoxicity and increased risk of delirium), or lidocaine (due to associated hemodynamic changes).

### 3.5. Drug Selection: Conmbinations and Rotation of Intravenous Sedative Drugs

The sedation of critically ill patients must be constantly adapted to the needs and objectives of their treatment. Modern intravenous agents are the routine choice due to their short duration of action and the fact that they produce few or no persistent metabolites. However, problems may arise; for example, the level of sedation provided may be insufficient or dose accumulation may occur, leading to prolonged weaning times or the development of serious complications such as PRIS. There is also the possibility of withdrawal symptoms when the drug is stopped. Thus, it is necessary to have other treatment options available and use these either in rotation or combination alongside adequate analgesia.

SPICE III is the most recent large-scale study comparing the effects of DEX and propofol in critically ill patients subject to mechanical ventilation [39]. In this study, high doses of both drugs were administered (1.0 µg/kg/h of DEX; propofol: between 50 and 200 mg/h), meaning there was a low requirement for antipsychotics or midazolam. Comparing patient outcomes depending on the drug they received, although the differences were not significant, patients receiving DEX had a higher incidence of adverse side-effects (9.6% vs. 1.8%), principally hypotension and bradycardia, although treatment was not required for these issues in most cases. Patients administered DEX also needed additional drugs to reach the desired level of sedation, a result found in a study on two drugs, MIDEX and PRODEX [40] where, respectively, 43.8% and 72.5% of patients required intravenous rescue medication.

A secondary Bayesian analysis of the SPICE III data [41] showed a highly reduced rate of mortality at 90 days for some patient groups, specifically non-surgical patients aged under 65 years sedated with a combination of DEX and propofol, where doses of the latter were increased to produce adequate sedation. Furthermore, this trend was even greater where DEX rather than propofol was increased to provide full sedation. What is undoubtedly true, then, is that the use of both drugs in combination can reduce the need to escalate the dosage of a single agent, thus reducing the risk of adverse dose-related side-effects from either drug. However, the impact on mortality of combining these sedatives and their relative dosage remains unknown, and similarly, the nature of their pharmacodynamic interaction and the influence of patient age are yet to be fully understood. These considerations, along with the increased incidence of hypotension observed with the combined use of these drugs in mechanically ventilated, critically ill patients [42] means that, currently, the routine combination of these intravenous sedatives is not advised. Although DEX and propofol are the agents most widely used with sepsis patients, the latter is considered more suitable due to the quality of infusion; this is particularly important in the first 48 h of treatment when requirements are highest and optimal levels of sedation are difficult to achieve with DEX alone. In addition, although in the case of patients who have undergone cardiac surgery it has been shown that sedation with DEX significantly reduced the time spent on mechanical ventilation and the development of delirium, it has not been possible to demonstrate similar benefits compared to other drugs in sepsis patients [43]. What can be said, nevertheless, is that sedation using DEX may be of relevance to that subgroup of sepsis patients with highest risk of delirium, as measured using the predictive scales available.

Finally, there are special considerations in selecting and dosing these two drugs for obese patients, specifically if a patient has a BMI over 40 kg/m^2^. In such cases, adjustments to the dosage protocol are required: propofol and DEX should both be administered in doses considered adequate for the patient’s ideal weight, while antipsychotics such as Haloperidol and Quetiapine should be administered in standard doses [44].

## 4. The Role of Inhaled Sedation Agents

Where patients develop high serum CPK (>5000 IU/L), hypertriglyceridemia > 400 mg/dL, or elevated levels of creatinine or transaminases, this constitutes a trigger for initiating a rotation regime of propofol with other drugs, or for considering inhalation agents as an alternative.

Currently, there are several devices available that would allow the administration of volatile anaesthetics in ICUs: Sedaconda^®^ (Sedaconda-ACD. Sedana Medical, Danderyd, Sweden) and the MIRUS^®^ system (Pall Medical, Dreieich, Germany) [45]. Both devices use a so-called *reflector* to enable the re-use of exhaled anaesthetic in the subsequent inspiration cycle. The Sedaconda can be used with two anaesthetics, isoflurane and sevoflurane; meanwhile, the Mirus is suitable for administering not only these two, but also desflurane.

The Sedaconda is the more rudimentary device, with only one rate of infusion determined by the electric syringe pump used to supply the anaesthetic agent. The Mirus system, in contrast, permits the infusion rate to be adjusted through the automated control of end tidal concentration targets. It must be said, however, that the Sedaconda has the more efficient reflector system [46].

With respect to the selection of anaesthetic gas, isoflurane is deemed most suitable for prolonged use in ICU settings. This result comes from a 2021 study [47] comparing isoflurane and propofol in a non-inferiority trial with 301 patients. The corpus comprised a heterogeneous group of patients admitted to ICU, and the study did not focus on sepsis patients. The findings show decreased extubation times and lower opioid use for isoflurane compared to propofol and, crucially, provide evidence for the safety of the long-term use of isoflurane, without the risk of more adverse effects than would be expected with propofol. Sevoflurane is generally used where there is an expectation of prolonged anaesthesia, but for periods no longer than 48 h, while desflurane use is minimal at present due to both the difficulty of administration and its high cost [48].

Beyond certain safety-at-work concerns which will not be dealt with further here, the limitation on the clinical use of inhalation agents is renal toxicity. These agents are metabolised in the kidney, and an upper limit on the levels of inorganic fluorides of 50 µmols/L has been established through experience with methoxyflurane. However, later studies have not been able to establish a correlation between this threshold and kidney damage [49,50]. Isoflurane is metabolised via conjugation to produce inorganic fluoride ions, which are excreted in urine, and here it must be highlighted that metabolism of this agent is much lower than for sevoflurane (0.2% vs. 5%); thus, in the case of isoflurane, fluoride toxicity is not relevant to the kidneys.

Clinicians will generally have most experience with the Sedaconda, device since it is far more widely used in the ICU than other devices, and thus, the following discussion will focus on the use of this device.

The objective in this section is to establish the therapeutic indications for inhalation sedation agents in terms of comorbidities and the clinical status of the sepsis patient. Nevertheless, it is important to stress that sedation cannot be considered as a therapeutic tool in itself, and according to the protocols set out in eCASH, the first step in care is providing effective analgesia, from which point we can go on to establish the minimum level of effective sedation. In the following, we consider the clinical contexts where inhalation sedatives are appropriate.

### 4.1. Special Circumstances for Consideration with Sepsis Patients

#### 4.1.1. Sepsis Patients with ARDS

Pulmonary involvement is frequent in sepsis patients, whether the origin of the sepsis is in the respiratory tract or otherwise. In fact, the abdominal region is the second most common sepsis origin associated with this complication [51].

In vivo porcine models of endotoxin-induced respiratory distress have demonstrated improved oxygenation with the use of sevoflurane in comparison to propofol [52], and these same models have also shown evidence of a reduction in the levels of systemic pro-inflammatory cytokines [53]. It is thought that the physio-pathological explanation for the observed beneficial effects of volatile agents in sepsis patients is based on their immunomodulating effects, specifically the immunosuppression of neutrophils, macrophages, and T-, B-, and NK-cells [54]. In humans with moderate or severe respiratory distress, improvements in the values of pO_2_/FiO_2_ are seen 48 h after the administration of sevoflurane, in addition to decreases in the levels of epithelial cell damage markers; however, no differences are seen in terms of mortality when compared to patients treated with midazolam [55].

Where the etiology of respiratory distress is a primary infection of the respiratory tract, it is of note that inhalation agents have been shown to have an antibacterial effect against *Streptococcus pneumoniae* and *Haemophilus influenza* [56]. The antimicrobial action of these agents has also been demonstrated when applied in liquid form against drug-resistant bacterial strains infecting wounds and it is speculated that their inhalation—particularly of isoflurane—could protect against ventilator-associated pneumonia infection [57]. Furthermore, inhalation agents, especially sevoflurane [58], are known to be bronchodilators and have been used very effectively in asthma patients, and they have been proposed as a therapeutic option in sepsis patients with respiratory distress [59]. In a slightly different context, recent work in patients with COVID-19 infection demonstrated that sedation with isoflurane is associated with improvements in arterial oxygenation, in addition to a lower consumption of opioids [60].

The negative aspects of inhalation agents in cases of ARDS are essentially those associated with increased dead space in the devices used to administer the drug. The problem of dead space is inherent to both the Sedaconda and the Mirus, and to resolve this issue, in the case of the Sedaconda, there are two improved devices: the L- and the S-versions. The most sophisticated of these is the S-version, which has dead space equivalent to the dimensions of a conventional HME filter, that is, a minimum in-use tidal volume of 200 mL. Comparing the S- and the L-devices in conditions of normal use, gas monitoring shows the former gives a significantly lowered pCO_2_, with only a slightly reduced reflection capacity in the activated carbon membrane [61]. Without doubt, then, the Sedaconda-S is the device of choice for patients with ARDS.

At present, clinical trials are ongoing comparing sevoflurane and midazolam in patients with respiratory distress [62]. The corpus in question comprises an estimated 700 patients, and the trial’s aims are firstly to assess ventilation-free days and secondly to record mortality to generate a set of guidelines for the use of inhalation sedation agents.

#### 4.1.2. Sepsis Patients with Myocardial Dysfunction

It is well known that myocardial dysfunction in sepsis patients is linked to a 20 to 50% increase in mortality [63]. The etiology of this condition is unknown; however, it is related to three mechanisms, each of which has a different weight depending on the patient: myocardial ischemia, direct damage from inflammatory mediators, and sepsis-related mitochondrial dysfunction [64]. Clinical presentation includes ventricular systolic or diastolic dysfunction and dilation.

One of the most important and consistently present mechanisms in myocardial dysfunction is ischemia and, in cases of septic shock, high levels of myocardial damage markers are observed, leading to a phenomenon known as myocardial hibernation. This is an adaptive phenomenon to protect the body from the adverse effects of ischemia, and is conceptually similar to cardiac postconditioning. In such cases, inhaled sedation agents have a particularly beneficial role; for example, mouse models have linked the use of nitric oxide with sevoflurane to reductions in myocardial damage [65]. Regarding the elevation of myocardial damage markers, here, sevoflurane has been shown to have protective properties in addition to other advantages, specifically, compared to intravenous sedation, it is linked with a lower use of vasopressors in cardiac surgery [66].

There is, nevertheless, a lack of evidence to recommend the use of inhalation sedatives in sepsis-related myocardiopathy. Rather, it is indicated theoretically on one hand for physio-pathological reasons, and on the other because it does not have the same undesirable inotropic effects associated with propofol. One published meta-analysis of propofol using in a corpus of 30,757 patients showed this drug is linked to increased mortality rates, especially in the sub-group of cardiac surgical patients, where the comparison drug is an inhalation sedation agent.

In this way, in cases of sepsis where the patient has a history of myocardial ischemia, the choice to use an inhalation sedation agent is justified. In fact, due to their cardioprotective and vasodilatory effects, several guidelines, such as those of the American College of Cardiology or American Heart Association [67], place volatile agents in the class IIA category for anaesthesia maintenance in patients with myocardial ischemia undergoing non-cardiac surgery. This recommendation for intraoperative procedures can be translated to the ICU once the technology becomes available to administer and monitor inhalation agents in the same way as in the operating theatre. However, at present, there are no recommendations for the use of volatile agents to be used for their cardioprotective effects.

#### 4.1.3. Sepsis-Associated Encephalopathy

The central nervous system (CNS) is one of the first organs affected in sepsis, and its clinical manifestation is so-called sepsis-associated encephalopathy (SAE). The incidence of SAE is about 50% [68]. Its pathophysiology comprises neuroinflammation, vascular changes, and metabolic failure leading to tissue lesions. The centres involved in autonomic controls, arousal, awareness, and behaviour are particularly affected, accounting for the clinical features of SAE varying from sickness behaviour to consciousness impairment (i.e., ranging from delirium to coma) [69].

SAE is defined by the combination of extracranial infection with clinical signs of neurological dysfunction. SAE clinical manifestations encompass the impairment of awareness, which ranges from delirium (50%) to coma (46%) [70]. Sepsis-associated delirium (SAD) is one of the symptoms of SAE.

Delirium frequently develops in the ICU, and the prevalence of each phenotype defined by clinical risk factors has been examined. Among 1040 patients, 71% were diagnosed with delirium at some point during their ICU stay: sedative-associated, hypoxic, septic, metabolic, and unclassified delirium in 64, 56, 51, 25, and 22%, respectively [71].

There are numerous risk factors for SAD and SAE: older age, a history of chronic alcohol abuse, neurological disease, pre-existing cognitive dysfunction, the long-term use of psychoactive drugs, acute renal failure, hyperglycaemia, hypercapnia, hypernatremia, and Staphylococcus aureus bacteraemia are the more consistent [68]. Regarding pharmacological measures, the association between the use of benzodiazepines in patients on mechanical ventilation for more than 48 h an intra-hospital setting and one year mortality has been published [72]. Benzodiazepines are associated with the onset of delirium, and delirium is associated with a longer duration of mechanical ventilation and ICU admittance, as well as an increased risk of death, disability, and long-term cognitive dysfunction [73]. Thus, current guidelines [74] do not recommend the use of benzodiazepines except for in specific situations such as alcohol withdrawal syndrome.

Benzodiazepines are not recommended as first-line treatment for sedation; however, the SAD risk with alternative sedatives remains unclear [75]. A recent systematic review [76] evaluated the efficacy and safety of DEX versus other sedatives in mechanically ventilated adults in the ICU, and found a significant reduction in the risk of delirium, duration of mechanical ventilation, and ICU length of stay; however, DEX was not found to reduce the risk of death at 30 days compared with other sedatives.

Post-intensive care syndrome (PICS) is currently the focus of research on the long-term prognosis of patients after critical care, and consists of three major components: physical impairment, mental disorders, and cognitive dysfunction. Delirium is considered to correlate with PICS. Cognitive dysfunction was shown to be directly affected by delirium via neural damage. The occurrence of delirium was identified as an independent risk factor for long-term and permanent cognitive dysfunction in ICU patients, including those with sepsis [77]. Thus, interventions such us avoiding benzodiazepines that appear to have a beneficial effect on the development of delirium may contribute to improving the prognosis of PICS.

#### 4.1.4. Continuous Renal Replacement Therapy and Extracorporeal Membrane Oxygenation

The bibliography and recommendations in this area are very limited, especially with regard to Extracorporeal Membrane Oxygenation (ECMO). Midazolam is metabolised by CYP3A enzymes to form 1-OH-midazolam mainly and to a smaller extend 4-OH-midazolam. After hydroxylation, 1-OH-midazolam is subsequently metabolised to 1-OH-midazolamglucuronide as its major metabolite, which is renally excreted. Previous research showed that 1-OH-midazolam and 1-OH-midazolam-glucuronide have sedative potency compared to midazolam of 60–80% and 10%, respectively, and may therefore be responsible for unexpected prolonged sedative effects when used in patients with renal failure [78]. Midazolam and 1-OH-midazolam are not removed efficiently via continuous renal replacement therapy (CRRT), and approximately 43% of 1-OH-midazolam-glucuronide is removed via CRRT. CRRT modality, filter patency, and downtime of the CRRT circuit affect the clearance of the pharmacological active metabolite 1-OH-midazolam glucuronide [79]. Thus, the use of midazolam during CRRT should lead to incentives to decrease the midazolam dosage by as much as is feasible to mitigate adverse consequences of oversedation.

A retrospective study of 74 patients shows that patients who received propofol in CRRT required higher total dosage and longer delivery time to maintain a good sedation. The clearance rate of propofol and maintenance of a stable blood concentration were the same in CRRT group and no-CRRT group [80]. A previous study on 10 patients shows no increase in propofol consumption, but the initial introduction of the extracorporeal circuit was found to reduce plasma concentrations in the majority of patients [81]. With the use of propofol and in association with hypertriglyceridaemia, several cases of early CRRT circuit clotting have been reported [82]. The exact pathophysiology behind hypertriglyceridemia-induced CRRT clotting has not been fully elucidated. 

Preliminary investigations have demonstrated that the introduction of ECMO potentially leads to significant changes in the pharmacodynamics of drugs in three ways: (1) drug sequestration by the circuit; (2) increased volume of distribution; and (3) altered drug clearance. Significant circuit drug sequestration is suggested for propofol, DEX, and midazolam, with higher daily doses recommended than usual [83]. However, field studies carried out do not show an increase in the need for sedatives, especially propofol and DEX [84].

With these data, the only firm recommendation that can be made in this context is that of close clinical monitoring.

## 5. Conclusions and Future Directions

There is already significant awareness among clinicians of the importance of appropriate sedation and analgesia in ensuring the best patient outcomes. This is clear from changes in practice, such as to the avoidance of benzodiazepines for critically ill patients due to their associated high mortality risk [72]. Nevertheless, different members of the ICU team caring for a particular patient may have varying priorities with regards to the objectives of sedation [85], and as a result it is essential to ensure good communication between team members to achieve a consensus on the best approach for each patient. It is also necessary to consider the enormous workload for nursing staff associated with the infusion and dosing of sedoanalgesia; thus, the involvement and training of nurses in sedation procedures is essential, and they must be instructed not only in the infusion of the relevant drugs but also in the use of the various clinical scales for evaluating analgesia, sedation, and delirium.

The purpose of this article is to communicate the need to personalise sedoanalgesia depending on the patient’s individual pathology, as well as any comorbidities. For critically ill patients, as with any other medical procedure they undergo in ICU such as haemodynamic monitoring or ventilation, the personalization of sedoanalgesia is the only way forward if we wish to obtain the best outcomes for all patients. Indeed, we must go beyond simply avoiding the use of certain groups of drugs towards the concept of “objective-guided sedation”, taking full advantage of the therapeutic arsenal currently available to us.

With all of this in mind, Table 2 presents a range of recommendations for sedation in sepsis patients. These recommendations represent an attempt to summarise the information presented in this article, and are intended to be interpreted dynamically, with frequent re-evaluations being made in the light of every development in the patient’s condition. We are aware of the limitations of our proposal due to the restricted evidence available on which some of our recommendations are based; however, we willingly accept the risk of criticism if we succeed in awakening concern and instilling the notion that the best sedoanalgesic option should be sought on a case-by-case basis.

Based on current evidence, propofol should be the preferred sedation option in the majority of clinical scenarios, especially if we need deep sedation in ARDS with hypercapnia, and cardiomyopathy with the precaution of titrating the dose. Isoflurane should be considered as an alternative in cardiomyopathy and need of prolonged sedation. We must differentiate cardiomyopathy from the high need for vasopressors, where inhaled agents may show beneficial effects compared to propofol. We should take advantage of the anti-inflammatory properties of inhaled agents [53] on hypoxemic ARDS, making them the first choice of treatment. The agent of choice in light-sedation in patients with delirium should be DEX, using propofol in the case of agitation.

In the context of hepatic insufficiency and considering the poor reproducibility of pharmacokinetic models of propofol [86], we recommend looking for an alternative to it, considering the choice of isoflurane. In the case of renal insufficiency, sevoflurane is not indicated for use, and propofol and isoflurane should be considered the main options for prolonged sedation.

In prolonged sedation, the possibility of rotating propofol and inhaled agents should be considered to minimizes the side effects of prolonged infusion of both. With the current evidence and available options, benzodiazepines should be used in limited contexts, such as alcohol withdrawal.

As a final point, the number of publications concerning sedation increases exponentially each year, contributing to the rapid evolution of this area of clinical practice. Therefore, it is likely that many of the doubts concerning recommendations made in Table 2 will be resolved in time.

## Figures and Tables

**Table 1:** Evaluation and monitoring of analgesia, sedation and delirium. Summary of clinical scales and quantitative and semi-quantitative monitoring of sedoanalgesia. Visual Analogue Scale (VAS). Visual Numeric Scale (VNS). Nociception Level Index (NOL). Analgesia Nociception Index (ANI). Sedation Agitation Scale (SAS). Richmond Agitation Sedation Scale (RASS). Minimum Alveolar Concentration (MAC). Bispectral index (BIS). Confusion Assessment Method for the ICU (CAM-ICU). Intensive Care Delirium Screening Checklist (ICDSD). Critical Care Continuous Electroencephalogram (EEG).

Monitoring Sedoanalgesia		
Pain	Clinical scales	VAS (Self-reported VNS (Self-reported)
		BPS (not self-reported) CPOT (not self-reported)
	Analgesia monitoring	NOL ANI
Sedation	Clinical scales	SAS RASS
	Inhaled sedation	MAC-awake
	Sedation monitoring	BIS (only as an alternative)
Delirium	Clinical scales	CAM-ICU ICDSC
	Delirium monitoring	CCEEG or timely EEG

**Table 2:** Proposed recommendations for sedation in sepsis patients. * Evaluate as part of a rotation strategy; ** use if short-duration sedation planned; *** consider as adjuvant therapy. ^(1)^ Can limit the dead space in circuits. ^(2)^ Go to B if increase in vasopressor medication is indicated. ^(3)^ Biphasic elimination without renal toxicity; use especially where there is IAH-associated AKI. ^(4)^ Lowered metabolism, 60% dependent on hepatic flow and secondary effects. ^(5)^ Use in cases of agitation and/or aggression. ^(6)^ Elimination via ventilator. ^(7)^ No GABA activation, less risk of delirium than with Propofol. ^(8)^ May be used prophylactically depending on score for predicted risk of delirium. ETI: endotracheal intubation; PN: parenteral nutrition; PRIS: propofol-related infusion syndrome; ARDS: acute respiratory distress syndrome.

Clinical Condition Accompanying Sepsis							
	ARDS without Difficulty Ventilating	ARDS with Difficulty Ventilating	Myocardial Dysfunction	Renal Insufficiency	Hepatic Insufficiency	Delirium	Notes
Propofol	Option C *	Option A ^(1)^	Option A ^(2)^	Option A ^(3)^	Option B ^(4)^	Option A ^(5)^	Look out for signs of PRIS as from 48 h. Adjust for fats in PN.
Isoflurane	Option A	Option B *	Option B *	Option B *	Option A ^(6)^	Option B ^(7)^	Requires ETI. Monitor end-tidal volume if possible.
Sevoflurane	Option B **	Option C *^;^**	Option C *	Not indicated	Option C **	Option C **	
DEX	Not indicated if deep sedation required			Option C	Option C ***	Option A ^(8)^	May be administered to regulate sleep.
Notes	May be possible to administer intravenous propofol in conjunction with an inhalation agent where deep sedation is indicated.						

## Data Availability

Not applicable.
